# Comparison of the Efficacy and Safety of Extracorporeal Shock Wave Lithotripsy and Flexible Ureteroscopy for Treatment of Urolithiasis in Horseshoe Kidney Patients: A Systematic Review and Meta-Analysis

**DOI:** 10.3389/fsurg.2021.726233

**Published:** 2021-10-25

**Authors:** Xianyanling Yi, Dehong Cao, Pinghong You, Xingyu Xiong, Xiaonan Zheng, Tao Jin, Ge Peng, Hang Xu, Dazhou Liao, Qiang Wei, Hong Li, Lu Yang, Jianzhong Ai

**Affiliations:** ^1^Department of Urology, Institute of Urology, West China Hospital, Sichuan University, Chengdu, China; ^2^Department of Urology, People's Hospital of Deyang City, Deyang, China; ^3^Department of Endocrinology and Metabolism, West China Hospital of Sichuan University, Chengdu, China

**Keywords:** horseshoe kidney, urolithiasis, extracorporeal shock wave lithotripsy, flexible ureteroscopy, efficacy

## Abstract

**Background:** Urolithiasis is the most common complication of horseshoe kidney (HK), which can be treated by extracorporeal shock wave lithotripsy (ESWL), flexible ureteroscopy (FURS), and percutaneous nephrolithotomy (PCNL). When comparing treatments of ESWL and FURS, it is unclear which is more efficient and safe. The objective of this study was to compare the efficacy and safety of FURS and SWL for the treatment of urolithiasis in HK patients.

**Methods:** A systematic search of the Web of Science, PubMed, and EMBASE was performed in February 2021. Newcastle-Ottawa Scale (NOS) was used to assess the risk of bias in each study.

**Results:** Five studies published between 2008 and 2018 were synthesized in the present meta-analysis. The study revealed that FURS compared with SWL had greater initial and overall stone-free rates (SFRs). Risk ratios (RRs) were 2.46 (*P* < 0.00001) in initial SFRs, 1.36 (*P* = 0.02) in overall SFRs. No differences were found in the retreatment ratio, RRs were 0.49 (*P* = 0.43). In addition, no major complications were encountered, and all the complications were mild to moderate.

**Conclusion:** The study demonstrated that FURS and SWL are effective and safe treatments for patients with HK with stones (<20 mm). Moreover, FURS has greater clearance rates and lower complication rates than SWL.

## Introduction

Horseshoe kidney (HK) is the most common renal fusion anomaly, with an incidence range from 1/400 to 1/666 (1). HK occurs as a result of the abnormal fusion of the lower poles at the embryological stage. Consequently, the normal ascent and rotation of the kidneys are arrested, leading to malrotation with anterior displacement of the collecting system (2). Impaired drainage of the collecting system and ureteropelvic obstruction predispose the patients to urolithiasis and a higher incidence of infection (3, 4).

The most common complication of HK is urolithiasis, which is encountered at an incidence of 21–60% (2). For early-phase treatment of urolithiasis in HK, open-operative approaches were mainly taken. However, minimally invasive surgery and non-invasive treatment are gaining popularity for smaller incisions, fewer complications, less postoperative pain, and shorter length of hospital stay. Extracorporeal shock wave lithotripsy (ESWL), flexible ureteroscopy (FURS), and percutaneous nephrolithotomy (PCNL) are the currently available methods for treating calculi in HK. There have been no guidelines or standard criteria for the selection of the favorable approach for the treatment of calculi in HK, especially for <20 mm renal stones (5). PCNL is the first choice therapeutic option for stones larger than 2 cm (5, 6). Despite the fact that PCNL has the higher success rates, the risk of complications (complication rates 83%) cannot be neglected due to the invasive nature of PCNL (7, 8). Nevertheless, the latest study demonstrated that PCNL in patients with HSK is safe and effective with a low complication rate (17%) (9). Additionally, laparoscopic lithotripsy, including retroperitoneal and transperitoneal, seems to be safe and effective for patients with HK with a limited number (*n* ≤ 3) of 20–40 mm renal stones, but these patients require prolonged hospitalization (10). Inevitably, it is a less invasive approach being more preferable. Uncomplicated and small (<15 mm) calculi can be treated non-invasively by SWL, which is a common and well-tolerated procedure for HK with stones (11–13). Additionally, with the development of technology, FURS has shown promising prospects of urolithiasis therapies with high stone-free rates (SFRs) and low complication rates, especially in moderate- or small-sized stones (14, 15). Whereas, there is no common consensus on which approach is most appropriate.

Recently, some studies reported the experience of the therapeutic effects of SWL and FURS. Accordingly, the objective of this study is to compare the efficacy and complications of FURS with SWL in the treatment of HK with stones. Ultimately, we can provide guide treatment selection for the treatment of HK with stones.

## Methods

### Literature Search and Data Extraction

This systematic review of the literature was performed in February 2021 using the Web of Science, PubMed, and EMBASE. Preferred Reporting Items for Systematic Reviews and Meta-Analyses (PRISMA) statement was followed. The comprehensive search of the studies was carried out independently by two investigators using the following string terms: (“HK” OR “fused kidney”) AND (“stone” OR “calculus” OR “calculi”). The search was limited to English-language literature, and no date restrictions were applied.

The population, intervention, comparator, outcome, and study design (PICOS) approach was used to define the study eligibility. Inclusion criteria were (P) patients were diagnosed as HK with urolithiasis (the diameter of the stone is <2.0 cm); (I) undergoing FURS; (C) in which SWL was performed as a comparator; (O) evaluating the following outcomes: initial SFRs, overall SFRs, retreatment ratio, and complication; (S) randomized controlled trials (RCTs), non-zRCTs, prospective observational studies, or retrospective observational studies. The exclusion criteria include the following: <2 treatment arms; non-English publications without an English abstract; and studies with unavailable or incomplete outcome data. Meanwhile, editorial comments, letters to the editor, case reports, and meeting abstracts were also excluded.

All database results were imported into an EndNote X7 reference manager prior to screening, and then duplications were removed. Titles, abstracts, and full-text articles were screened by two authors. Additionally, data extraction was carried out by the reviewers independently. The data we extracted included the following: setting, date, study design, participant demographics, baseline characteristics, intervention details, and outcomes.

### Quality Assessment

The studies we included were assessed by Newcastle-Ottawa Scale (NOS). Studies with NOS scores <5 were considered as low-quality studies, 6–7 were considered intermediate-quality studies, and 8–9 were considered as high-quality studies.

### Statistical Analysis

Meta-analysis was conducted with Cochrane Collaboration Review Manager software (RevMan v.5.3.0). Continuous variables were presented as mean ± SD or the minimum-to-maximum range, whereas categorical variables were expressed as percentage or number of individuals. Treatment results were calculated using the Mantel-Haenszel method and evaluated by risk ratios (RRs) and 95% CIs. When RR is more than 1.0, it indicates a greater likelihood of SFR in the FURS group. The two-tailed test was used to assessed statistical significance (test level α = 0.05), and *P* < 0.05 was considered significant.

Assessment of heterogeneity will be assessed by chi-squared test and *I*^2^ test, *P* < 0.1 and *I*^2^ > 50% indicated heterogeneity. The fixed-effects models were used for calculation when no significant heterogeneity was observed. Sensitivity analysis was conducted by excluding studies from the analysis one by one, and a random-effects model instead of a fixed-effects model was also used to test the robustness of the meta-analysis results. A formal assessment of publication bias was unable to be evaluated due to the limited number of included studies.

## Results

### Characteristics of Eligible Trials

**Figure 1:**
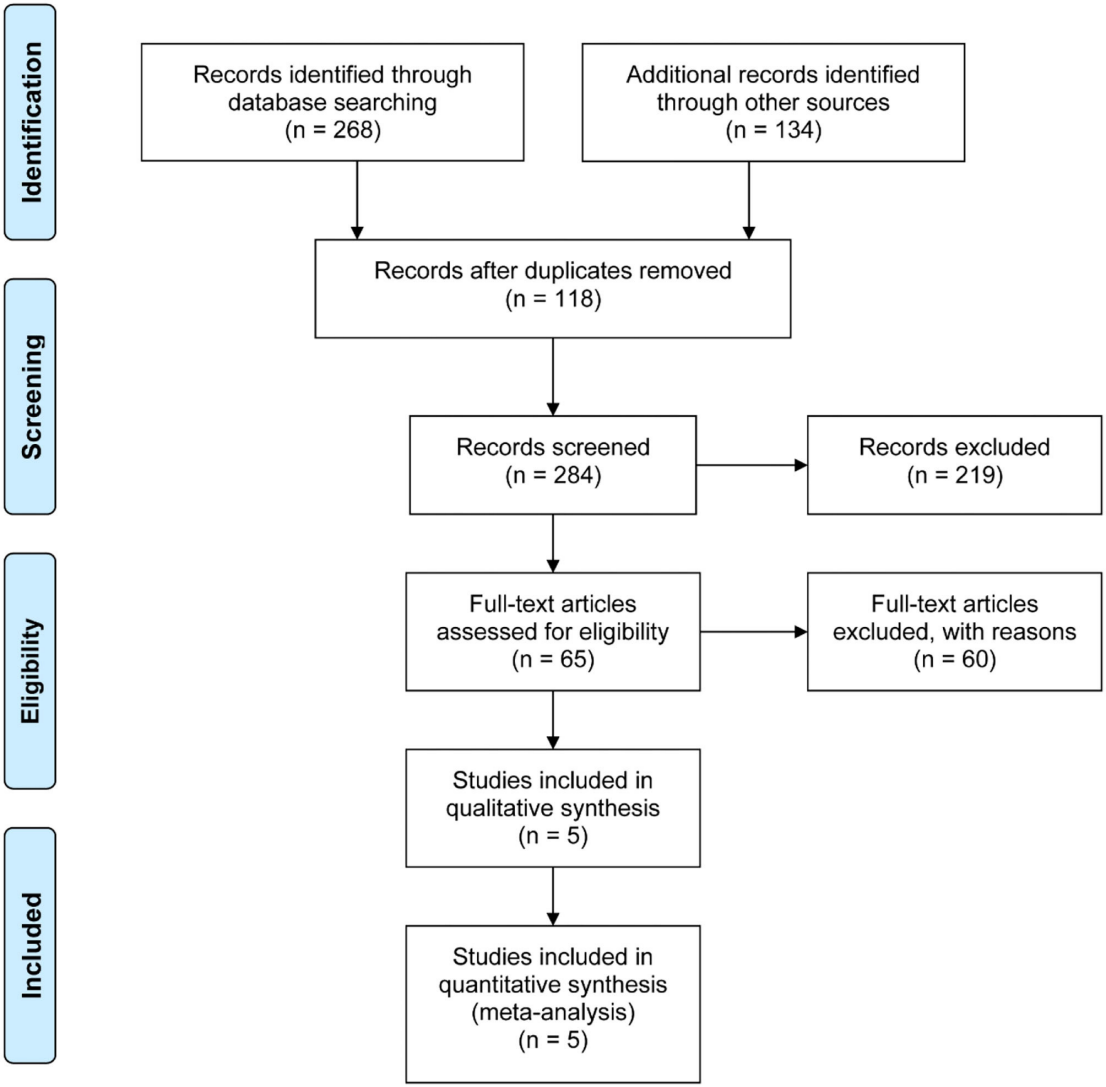
Preferred reporting items for systematic reviews and meta-analysis flowchart.

The search strategy identified 284 records and 65 remained eligible for inclusion based on screening. Of the 65 full-text articles assessed for eligibility, a total of five cohorts were invited to participate (Figure 1) (16–20). Sixty studies were excluded for the following reasons: 38 studies were excluded because of unavailability of data for statistics, five studies because of the study design, and 17 studies because they were single-arm studies. These included studies were published from five countries between 2008 and 2018.

The characteristics of the five studies were summarized in Table 1. All of the studies were retrospective, single-center studies. Patients were recruited from July 1991 to May 2015. As shown in Table 1, five studies get a 6–7 NOS, which means all studies we included were considered intermediate-quality studies and low risk of bias. A total of 134 patients were included in our study (87 males (65%) and 47 females (35%), and 66 in the FURS group vs. 68 in the SWL group). The mean stone size of patients was <20 mm. Moreover, calcium oxalate was the most common stone type, although it was not described in the studies of Gokce et al. (19) and Al Otay et al. (16). The definition of SFR was concordant in most of the studies we included, what are no residual fragments noted on follow-up imaging.

Table 1Characteristics of included studies.

**Table:** 

**Study**	**Country**	**Study location**	**Period**	**Definition of SFR**	**NOS**
Al Otay et al. (16)	Saudi Arabia	Single center	2000–2012	No residual stones left behind on CT scan	6
Blackburne et al. (17)	US	Single center	2002–2015	No residual fragments noted on KUB, nephrostogram, or CT scan	7
Ding et al. (18)	China	Single center	2005–2014	No residual fragments noted on plain film and ultrasound	7
Gokce et al. (19)	Turkey	Single center	2003–2014	No residual fragments≥3mm in size in plain radiography, ultrasound and CT	7
Symons et al. (20)	India	Single center	1991–2008	No residual stones noted on follow-up imaging	6

**Table:** 

**Study**	**Treatment**	**Median Age, yr (range)**	**Gender (M/F)**	**Patient number**	**Numbers of renal moieties**	**Median Stone size, (range)**	**Stone location**	**Median duration of follow-up, (range)**	**Initial SFR**	**Overall SFR**
Al Otay et al. (16)	FURS	37 (2–78)	16/9	1	-	<10 mm	-	31.6 ± 24.1^*^ months (12–76)	-	-
	SWL			6	-	(10-20) mm	-		-	-
Blackburne et al. (17)	FURS	48.1 (29–28)	13/7	22	25	8.4 (2–25) mm	lower pole	20.5 months (0–118)	84%	100%
	SWL	32.5 (23–42)	0/2	2	2	4.5 (4–5) mm	-		50%	100%
Ding et al. (18)	FURS	42.9 ± 11.6^*^	14/4	18	20	18.9 ± 3.6 (339.6 ± 103.9 mm^2^)^*^	-	4 weeks	55.6%	88.9%
	SWL	36.6 ± 8.2^*^	9/2	11	12	11.9 ± 2.0 (110.6 ± 44.5^*^ (range, 63–205) mm^2^)	-	-	27.7%	72.7%
Gokce et al. (19)	FURS	44.2 ± 9.9^*^	18/5	23	-	17.1 ± 5.1^*^ mm	Lower pole 6; Pelvis and upper pole 17	(2–6) weeks	73.9%	73.9%
	SWL	42.8 ± 8.4^*^	32/12	44	-	16.8 ± 4.4^*^ mm	Lower pole 12; Pelvis and upper pole 32	(1–6) weeks	22.7%	47.7%
Symons et al. (20)	FURS	36.5 (7–60)	49/6	2	5	172 (63–281) mm^2^	Pelvis 1, multiple (in the pelvis, superior calyx, middle calyx and isthmus) 1	1 month	100%	100%
	SWL			5	6	149.2 (50–225) mm^2^	Upper calyx 1; Pelvis 3; Lower calyx 1		60%	80%

*FURS, flexible ureteroscopy; SWL, extracorporeal shock wave lithotripsy; SFR, stone-free rate*.

* *Mean ± SD*.

### Efficacy of Treatment

**Figure 2:**
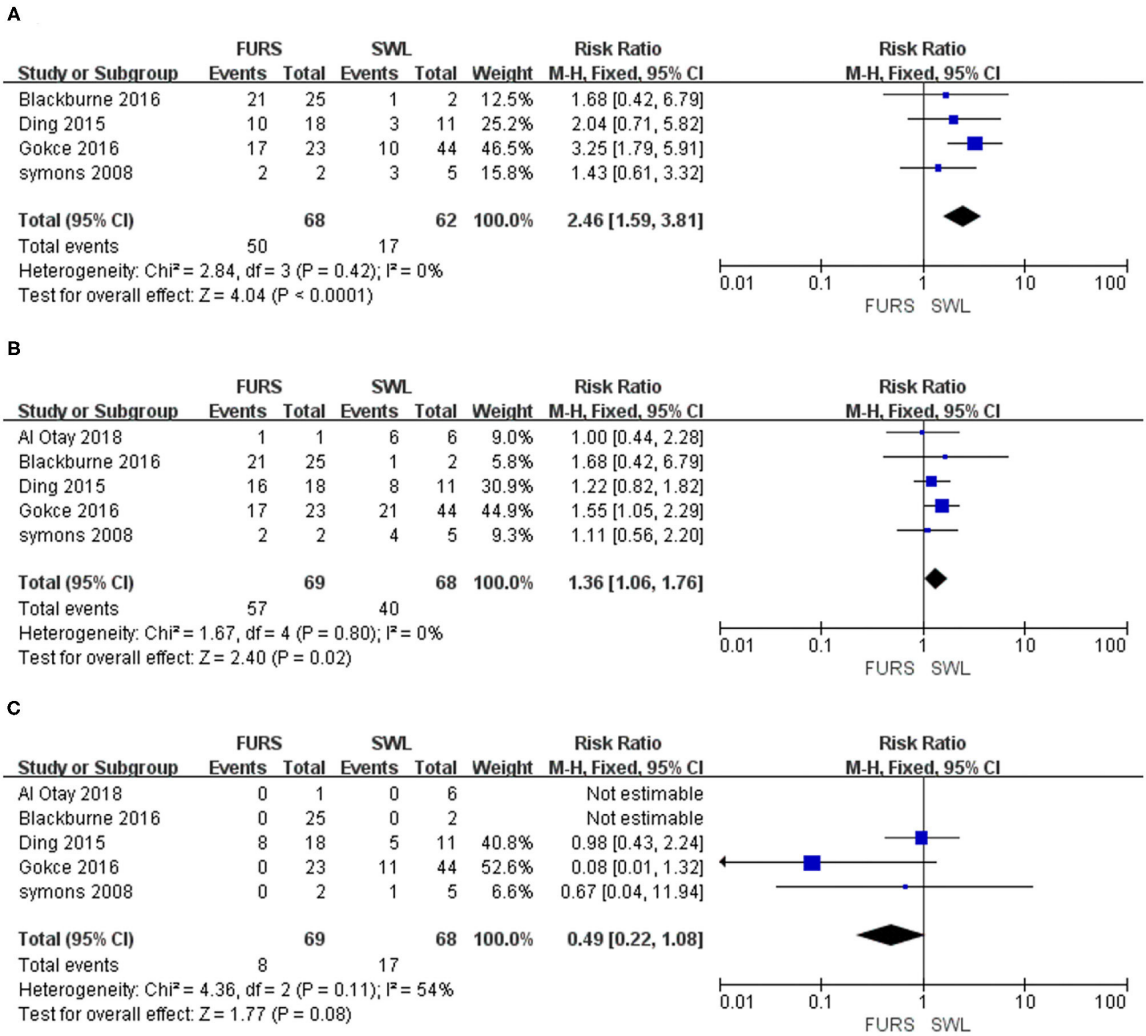
**(A)** Forest plot of initial stone-free rates for SWL vs. FURS. **(B)** Forest plot of overall stone-free rates for SWL vs. FURS. **(C)** Forest plot of retreatment ratios for SWL vs. FURS. SWL, extracorporeal shock wave lithotripsy; FURS, flexible ureteroscopy; CI, confidence interval; M–H, Mantel-Haenszel.

Results of the efficacy of surgery are based on all 134 patients from FURS and SWL. The rates of initial SFRs were ranged from 55.6 to 100% and 22.7 to 60% in FURS and SWL groups (Table 1). Additionally, there were statistically significant differences between the two groups overall, and RRs were 2.46 (95% CI 1.59, 3.81, *P* < 0.0001, *I*^2^ = 0; Figure 2A). Repetitive treatment was used in some patients with low stone clearance rates. Moreover, the rates of overall SFRs were ranged from 73.9 to 100% and 47.7 to 100% in FURS and SWL groups, respectively. Similarly, the overall SFRs of the FURS group were significantly better than that of the SWL group (RR = 1.36, 95% CI 1.06–1.76, *P* = 0.02, *I*^2^ = 0; Figure 2B). Nevertheless, there was no evidence that the retreatment ratio of the two groups was different (RR = 0.49, 95% CI 0.22–1.08, *P* = 0.11, *I*^2^ = 54%; Figure 2C). A sensitivity analysis of all studies indicated consistent results (Supplementary Figures 1–4).

### Complications

In total, 41 patients developed complications after FURS and SWL. The renal colic episode, which was the most common complication encountered, was presented in 20 patients; patients in the FURS group accounted for most of the total number. Five patients had a fever, and six patients had hydronephrosis. While hematuria was observed in nine patients, and the perirenal hematoma was observed in one patient. Additionally, no serious complications have been reported in the studies we included. On the whole, the rate of complications among patients of the SWL group was higher as compared to FURS groups (36.8 vs. 24.2%; Table 2). Whereas complications of FURS and SWL were not reported in the studies by Al Otay et al. (16), Gokce et al. (19), and Symons et al. (20), we failed to perform a further meta-analysis.

**Table 2:** Complications of FURS and SWL.

**Complication**	**FURS (*n* = 66)**	**SWL (*n* = 68)**
Renal colic episode	3	17
Fever	4	1
Hydronephrosis	6	0
Hematuria	3	6
Perirenal hematoma	0	1
Total	16 (24.2%)	25 (36.8%)

*FURS, flexible ureteroscopy; SWL, extracorporeal shock wave lithotripsy*.

## Discussion

In this meta-analysis, we compared the efficacy and complications between FURS and SWL in HK stones. Our findings suggested that patients with HK treated with FURS showed higher SFRs and lower complication rates than SWL. However, no statistical differences in terms of retreatment ratio were observed between the two groups.

To our knowledge, unique anatomical features of the HKs lead to impaired renal pelvic drainage, which accelerates the formation of stones. Moreover, patients with HK with stones may be troubled with some complications, such as pyelonephritis, hydronephrosis, and pyonephrosis. The therapy of HK stone is more difficult than the stone in a normal anatomical kidney. Published data have clearly shown that PCNL was an efficient mini-invasive stone removal procedure for patients with HK, particularly in the case of large stones (with a diameter larger than 2 cm) (9, 21, 22). However, since the high risk of complication associated with PCNL was performed for HK stone, SWL and FURS have great application prospects for relatively efficient and safe (8, 20).

Currently, SWL is one of the most commonly used treatments for urolithiasis, and stones smaller than 1.5 cm in patients with HK without ureteropelvic junction obstruction could be removed successfully with SWL (usually need repeat sessions) (23). Aside from this, Kirkali et al. (13) and Serrate et al. (24) reported that SFRs of SWL were between 28 and 80%, which was lower when compared to patients with normal kidneys. FURS was gradually applied to the management of renal stones in HSKs since 2005, and 75% (three of four) patients were complete stone clearance in the report of Weizer et al. (15). Breda et al. (25) demonstrated that the SFR is not the same across the size of the stone, and the SFR was higher in patients whose intrarenal stone burden <2 cm. Likewise, Molimard et al. (26) found that SFR was 53% after one session of FURS, and it rose to 88% after an average of 1.5 sessions (the mean stone size was 16 mm). Surprisingly, not only no complications were observed, but also the efficacy of FURS was similar to PNCL. Lavan et al. (27) published a more recent review on the outcomes of ureteroscopy for stone disease in anomalous kidneys, and they reported that patients who underwent FURS got good stone-free rates with a low risk of major complications, although the technic is challenging. This evidence indicated that SWL and FURS could be a feasible and safe alternative in patients with HK with calculus. As state in the latest European Association of Urology (EAU) guidelines, SWL can be used in patients with HK with stones, but the passage of fragments might be poor, while FURS can achieve acceptable SFRs (28). Furthermore, there is a debate about which therapy is more favorable for patients with HK with stones.

In our study, the SFRs of FURS and SWL were similar results to previous studies. As for the stone size of small to moderate, the removal of stone had an obvious effect. Nevertheless, both initial and overall SFRs were lower in the group of SWL, perhaps owing to the anatomic constraints of the HK, which makes stone fragments pass pelvis and ureter difficult. Compared with FURS, the main advantages of SWL are that general anesthesia does not require, and it is less expensive. Although the effect of FURS was stronger and statistically significant, the surgical procedure is complicated for flatter renal pelvis and narrower intrarenal space of HK kidney, which increases the difficulty of navigating and deflecting ureteroscope inside the kidney (29). Atis et al. (30) reported that the location in the lower pole was one of the factors for clearance failure of FURS in HK. Blackburne et al. (17) also arrived at similar conclusions. The SFR was lower for HK stones located in the lower pole. However, the clearance of stones at different locations was not clear in the other studies we included. Moreover, some patients receipted reoperation after an initial failed procedure and achieved the stone-free status, but there were no statistically significant differences between the two treatments.

As for complications, we found that the complication rates were comparable to previous studies. The abnormal anatomical kidney increases the difficulty of operation as well as prolongs the procedure time, which may lead to a higher rate of complications. Fortunately, no major complications were encountered, and all the complications were mild to moderate. Still, the different follow-up duration of studies should be taken into account, and long-term efficacy requires further investigation. Importantly, there were only two studies that reported complications, and the complications were not standardized by the Clavien-Dindo classification. Thus, more studies are required to confirm the safety of SWL and FURS.

This study has some limitations. All included studies were retrospective single-center studies, and the number of included patients was relatively small. Additionally, for the individual studies, baseline differences of each study might confound the results, especially the age of patients. The definition of SFR was different in included studies, as does the imaging tools used to assess SFR. However, there was no significant heterogeneity in this study, and our findings are reliable. And sensitivity analyses indicated that these pooled results were robust. Further, additional larger-scale studies are needed to confirm the findings of the present study.

## Conclusion

This study indicates that FURS and SWL are effective and safe treatments for patients with HK with stones. Moreover, FURS has greater clearance rates and lower complication rates than SWL. However, large- and high-quality RCTs are warranted to confirm the results of this study.

## Data Availability Statement

The original contributions presented in the study are included in the article/Supplementary Material, further inquiries can be directed to the corresponding author/s.

## Author Contributions

JA, LY, and XY conceived the project and drafted the manuscript. XY and GP searched the databases. TJ, DC, XZ, and HX analyzed data. PY, QW, HL, XZ, and JA revised the manuscript. All authors read and approved the final version of the manuscript.

## Funding

This study was supported by grants from the National Natural Science Foundation of China (82070784 and 81974099) and a grant from the 1.3.5 project for disciplines of excellence, West China Hospital, Sichuan University (ZYGD18011) to HL.

## Conflict of Interest

The authors declare that the research was conducted in the absence of any commercial or financial relationships that could be construed as a potential conflict of interest.

## Publisher's Note

All claims expressed in this article are solely those of the authors and do not necessarily represent those of their affiliated organizations, or those of the publisher, the editors and the reviewers. Any product that may be evaluated in this article, or claim that may be made by its manufacturer, is not guaranteed or endorsed by the publisher.
