# Supplementary material for: Comparison of the Efficacy and Safety of Extracorporeal Shock Wave Lithotripsy and Flexible Ureteroscopy for Treatment of Urolithiasis in Horseshoe Kidney Patients: A Systematic Review and Meta-Analysis
Source: Front Surg. 2021 Oct 25;8:726233. doi: 10.3389/fsurg.2021.726233 (PMC8572974; doi:10.3389/fsurg.2021.726233)
Supplement: Supplementary file 1 [file Data_Sheet_1.docx]

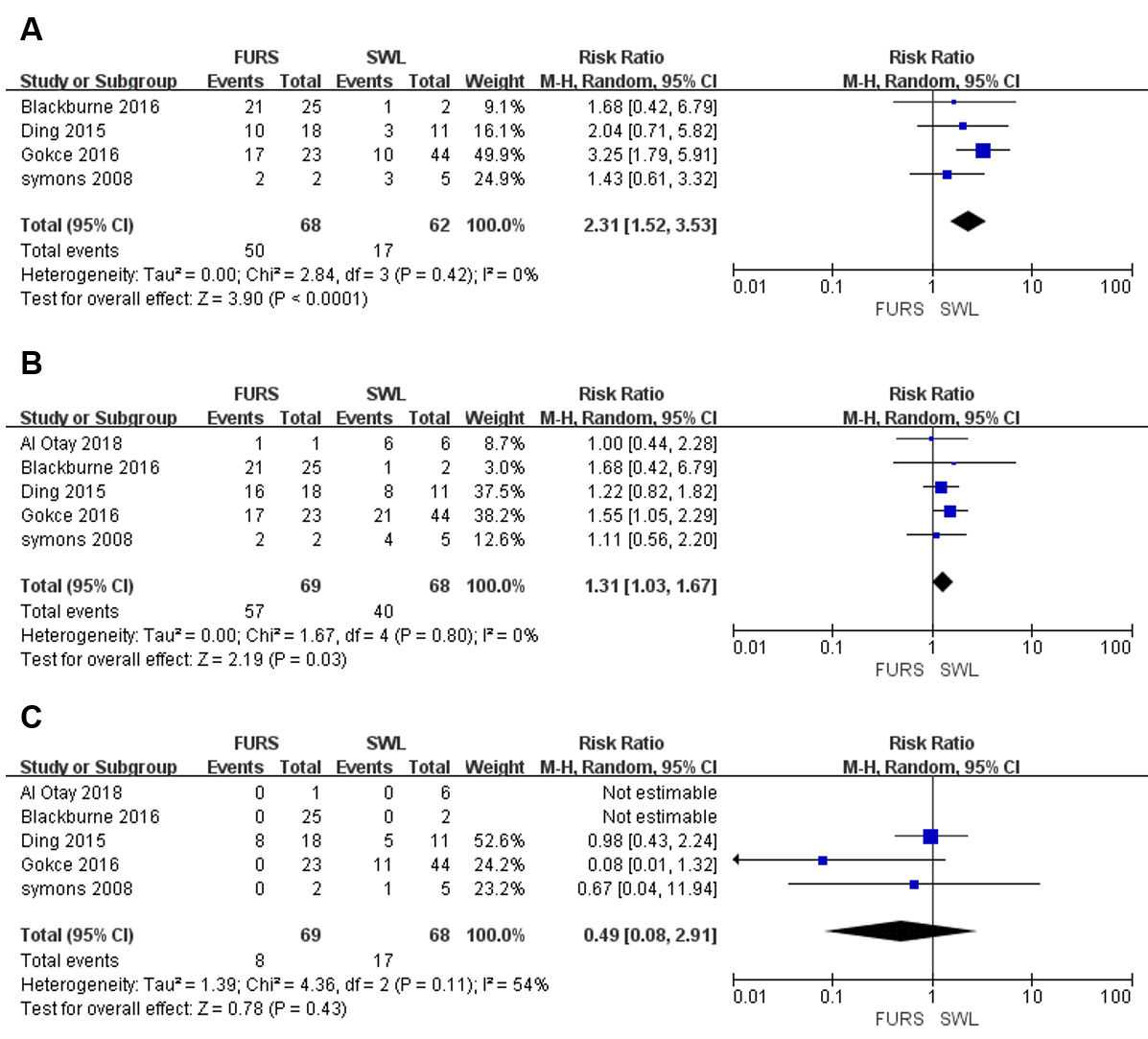


Supplementary figure 1. (A) Sensitivity analyses of initial stone free rates for SWL versus FURS. (B) Sensitivity analyses of overall success free rates for SWL versus FURS. (C) Sensitivity analyses of retreatment ratios for SWL versus FURS. SWL= extracorporeal shock wave lithotripsy; FURS= flexible ureteroscopy; CI= confidence interval; M-H= Mantel- Haenzel


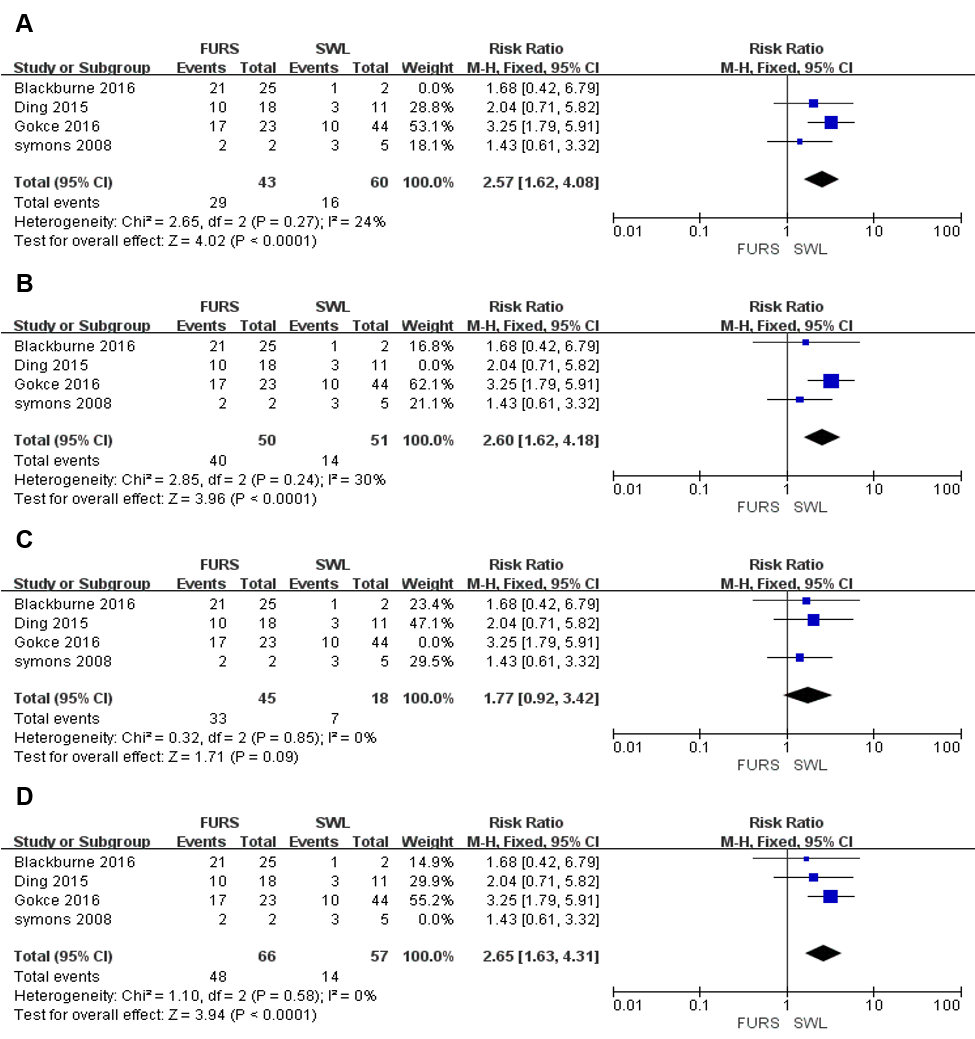


Supplementary figure 2. Sensitivity analyses of initial stone free rates for SWL versus FURS. SWL= extracorporeal shock wave lithotripsy; FURS= flexible ureteroscopy; CI= confidence interval; M-H= Mantel- Haenzel


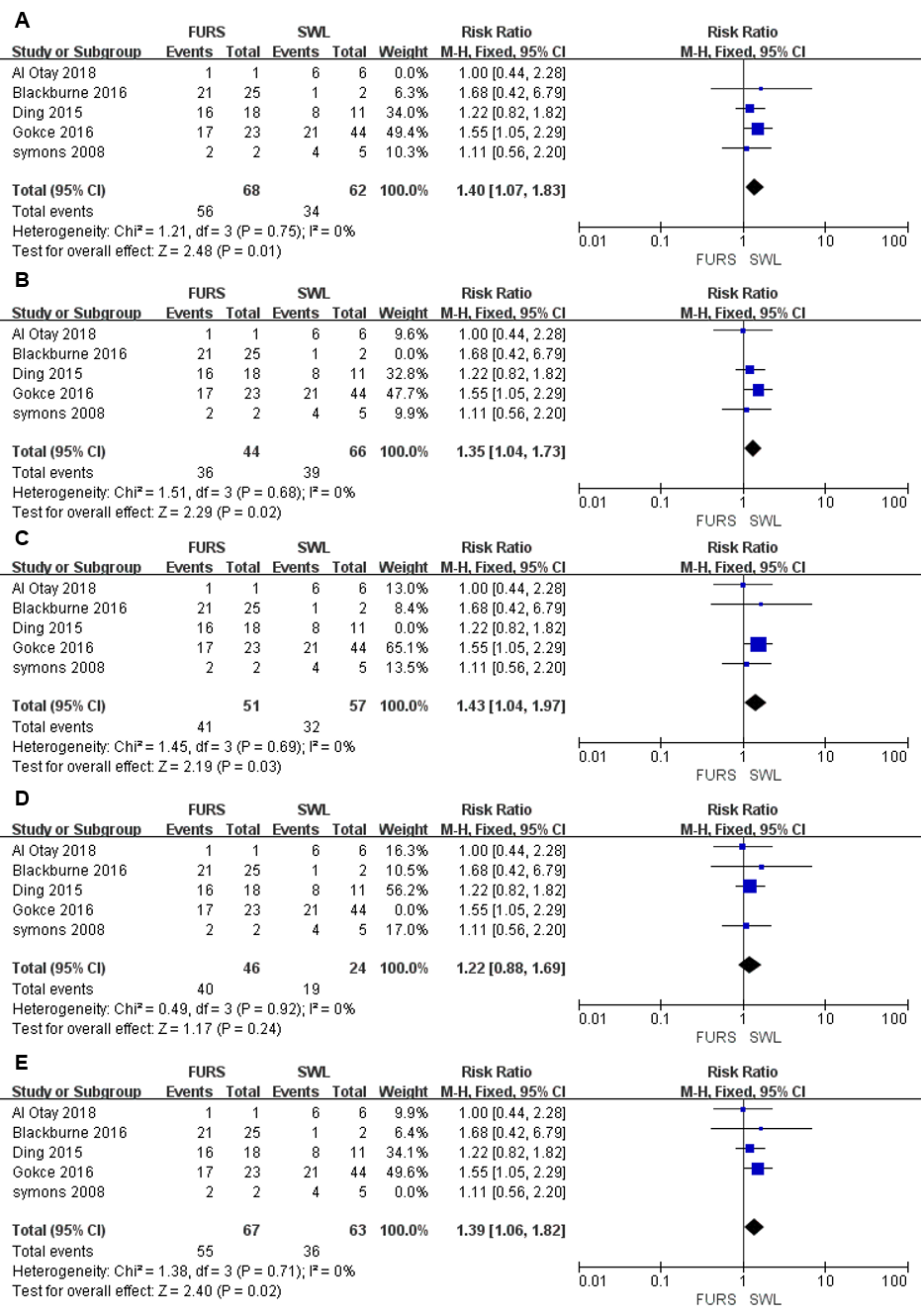


Supplementary figure 3. Sensitivity analyses of overall stone free rates for SWL versus FURS. SWL= extracorporeal shock wave lithotripsy; FURS= flexible ureteroscopy; CI= confidence interval; M-H= Mantel- Haenzel


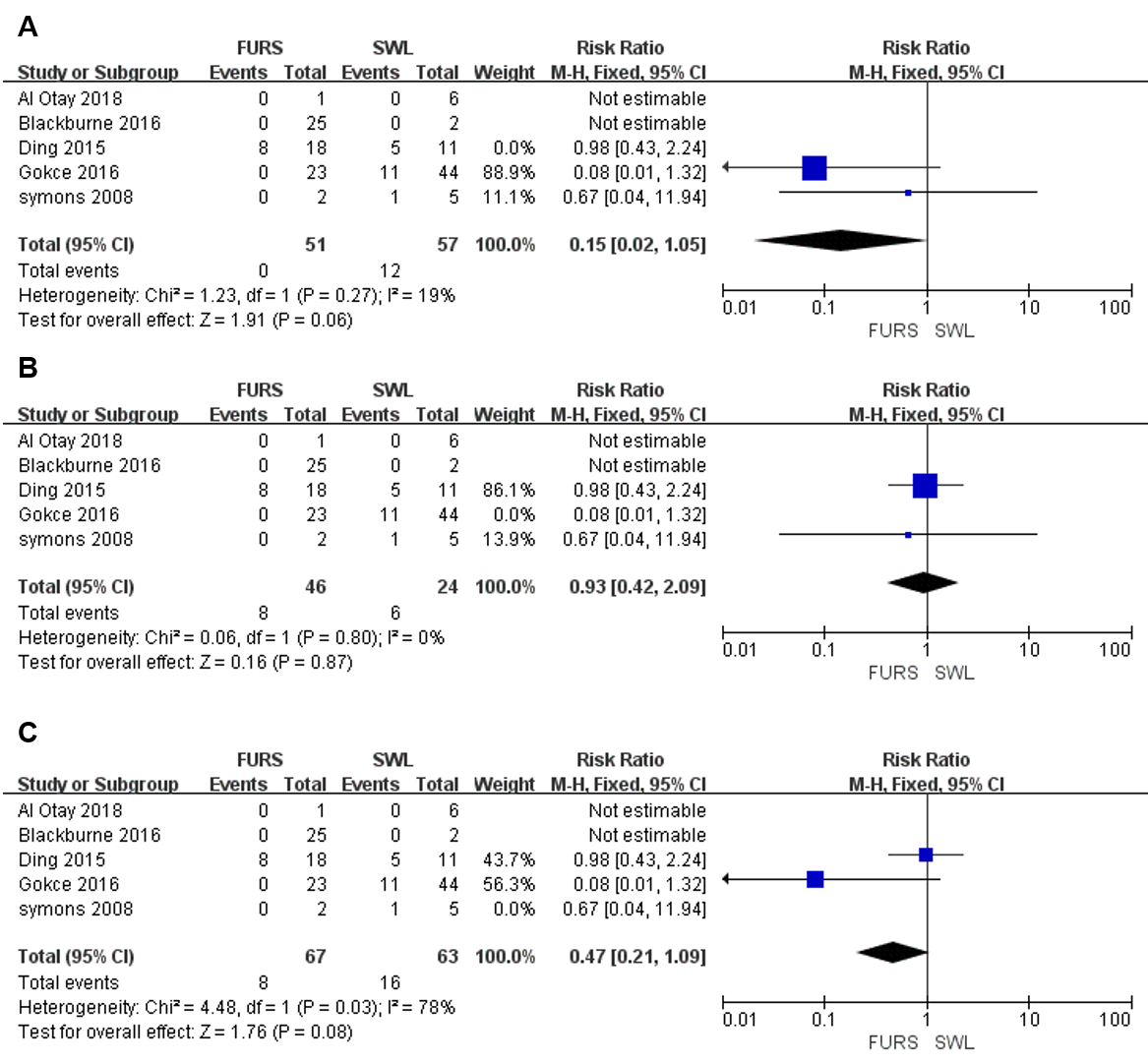


Supplementary figure 4. Sensitivity analyses of retreatment ratios for SWL versus FURS. SWL= extracorporeal shock wave lithotripsy; FURS= flexible ureteroscopy; CI= confidence interval; M-H= Mantel- Haenzel
